# Atypical Resting-State and Task-Evoked EEG Signatures in Children with Developmental Language Disorder

**DOI:** 10.3390/bioengineering13010119

**Published:** 2026-01-20

**Authors:** Aimin Liang, Zhijun Cui, Yang Shi, Chunyan Qu, Zhuang Wei, Hanxiao Wang, Xu Zhang, Xiaolin Ning, Xin Ni, Jiancheng Fang

**Affiliations:** 1 School of Instrumentation Science and Optoelectronic Engineering, Beihang University, Beijing 100191, China; liang-aimin@163.com (A.L.); zhang_xu@buaa.edu.cn (X.Z.); 2 Children’s Health Care Center, Beijing Children’s Hospital, Capital Medical University, National Center for Children’s Health, Beijing 100045, China; cuizhijun7788@163.com (Z.C.); faye0505@126.com (Y.S.); quchunyan72@163.com (C.Q.); wei9zhuang@aliyun.com (Z.W.); whanxiao6666@163.com (H.W.); 3 Beijing Children’s Hospital, Capital Medical University, National Center for Children’s Health, Beijing 100045, China

**Keywords:** developmental language disorder (DLD), electroencephalography (EEG), resting-state networks, event-related potentials (ERPs), neural biomarkers

## Abstract

Developmental Language Disorder (DLD) is associated with abnormalities in both intrinsic resting-state brain networks and task-evoked neural responses, yet direct electrophysiological evidence linking these levels remains limited. This study examined multi-level EEG markers in 21 typically developing children and 15 children with DLD across resting-state, a semantic matching task, and an auditory oddball task. Resting-state analyses revealed frequency-specific connectivity imbalances, reduced stability of intrinsic microstate dynamics, and atypical transitions between microstates in the DLD group. During the semantic matching task, DLD children showed weaker occipital P1 and N2 responses (100–300 ms) and lacked the right fronto-central difference wave (500–700 ms) observed in TD children. In the auditory oddball task, DLD children exhibited high-theta/low-alpha event-related desynchronization at left frontal electrodes (400–500 ms), in contrast to TD children. A machine learning framework integrating resting-state and task-based features discriminated DLD from TD children (test-set F1 = 70.3–80.0%) but showed limited generalizability, highlighting the constraints of small clinical samples. These findings support a translational neurophysiological signature for DLD, in which atypical intrinsic network organization constrains emergent neural computations, providing a foundation for future biomarker development and targeted intervention strategies.

## 1. Introduction

Developmental Language Disorder (DLD) is a prevalent and persistent neurodevelopmental condition that affects approximately 7% of children, leading to lasting impacts on academic, social, and occupational outcomes [1,2]. The recent consensus to replace “Specific Language Impairment” (SLI) with “Developmental Language Disorder” reflects a conceptual shift in the field. The term “Specific” was abandoned because it implied an isolated linguistic deficit, whereas “Disorder” acknowledges DLD as a multifaceted condition rooted in broader neurodevelopmental atypicalities [1].

This perspective reframes DLD as a disorder emerging from subtle, interacting deficits across cognitive domains [3]. Evidence shows that children with DLD often exhibit weaknesses in executive functions such as inhibitory control and working memory [4], along with impairments in low-level auditory processing, particularly in encoding rapid temporal cues [5]. Considering that language processing involves hierarchical, whole-brain computations [6,7,8], these findings may suggest that DLD involves both domain-general and domain-specific neural inefficiencies, highlighting the importance of investigating how intrinsic network dynamics contribute to inefficient language processing.

Resting-state electrophysiology provides a valuable way to detect key neural differences in DLD. One hypothesis proposes that DLD reflects atypical modulation of neural activity. However, findings based on traditional resting-state EEG measures of spectral power remain inconsistent. For example, one study of young children aged 4–6 years reported no group differences in power or hemispheric lateralization between children with DLD and their typically developing (TD) peers [9]. In contrast, another study of children aged 4–6 years found significantly lower alpha power in the DLD group over the left temporal and occipital regions [10]. A third study found that children with DLD showed reduced signal complexity and altered spectral parameters, including a lower aperiodic slope and differences in oscillatory power components compared with age-matched typically developing peers (ages 3–11) [11]. These findings suggest disrupted neural signal organization and atypical excitation–inhibition dynamics at rest, providing complementary evidence that static spectral power alone may not capture core neural alterations in DLD.

Recent advances highlight the importance of characterizing how brain oscillations are organized in time and space. Functional connectivity (FC) analysis extends beyond single-channel power to examine network coordination, revealing inefficient communication patterns in developmental disorders [12,13]. Complementarily, EEG microstate analysis offers a window into transient global network configurations—brief and quasi-stable topographies that reflect large-scale neural coordination [14,15]. Altered microstate dynamics have been reported in other neurodevelopmental conditions, such as autism [16], suggesting their potential relevance for DLD. Together, FC and microstate approaches support an “inefficient network” model, in which atypical intrinsic connectivity and unstable temporal dynamics undermine efficient neural communication. This intrinsic inefficiency provides the mechanistic link to task-evoked processes, predicting that DLD children may show atypical responses when engaged in cognitive tasks.

Event-related potentials (ERPs) provide a precise tool to trace these downstream consequences of intrinsic network inefficiency. Recent work has refined our understanding of Mismatch Negativity (MMN) alterations in children with DLD. Earlier reviews concluded that MMN amplitudes are generally reduced in DLD, reflecting impairments in pre-attentive auditory discrimination [17]. However, more recent studies have shown that MMN responses can appear typical when the acoustic differences between stimuli are sufficiently large in preschoolers with DLD [18,19]. Instead, group differences often emerge in later components such as the P300, which reflects attention allocation and inhibitory control [19]. This pattern indicates that the DLD deficit may lie not in automatic perception but in higher-order, resource-dependent processing.

At the linguistic level, semantic integration deficits are consistently observed in DLD, as reflected by attenuated or delayed N400 effects [20,21]. For example, preschoolers with DLD show a delayed N400 and atypical scalp distribution during sentence comprehension, suggesting slower or less efficient semantic mapping [21]. These findings converge on the view that DLD reflects a cascade of inefficiencies—from atypical modulation of neural activity to impaired task-driven processing.

However, the relationship between these two levels—resting-state network organization and task-based cognitive function—remains poorly understood. Current evidence is fragmented, with separate lines of research focusing on intrinsic dynamics (FC, microstates) or task-evoked responses (P300, N400). Integrating these perspectives may provide a more complete neurophysiological profile of DLD and improve data-driven classification. To date, no study has systematically tested the feasibility of combining multi-level EEG features for DLD identification.

The present study addresses this gap by exploring whether integrating resting-state and task-based EEG measures can differentiate DLD from TD children. The semantic and oddball tasks in the present study were specifically chosen to probe language related networks and auditory attention networks, respectively, which are hypothesized to be affected by inefficient intrinsic connectivity (resting-state). Specifically, we examined: (1) resting-state functional connectivity and microstate dynamics as markers of intrinsic neural organization; (2) task-based ERPs as indicators of resource allocation and semantic processing; and (3) the predictive potential of their integration using multiple machine learning models. We hypothesized that DLD children would show atypical resting-state networks, reduced task-evoked responses, and that a combined model would outperform single-modality classification, providing a first step toward a multi-level neurophysiological signature of DLD.

## 2. Materials and Methods

### 2.1. Participants

This study recruited 21 TD children (mean age 5.12 ± 0.87 years, range 4.0−7.0 years) and 15 children with DLD (mean age 5.23 ± 1.33 years, range 4.0−7.0 years). The age distribution between the two groups of children did not differ significantly (t = 0.29, *p* = 0.77). All were right-handed, native Mandarin speakers with normal or corrected vision and no history of neurological, hearing, or autism spectrum disorders. Children with parent-reported late talking or expressive language difficulties were evaluated for DLD. For children aged ≥6 years, cognitive ability was assessed using the Wechsler Intelligence Scale for Children Fourth Edition (WISC-VI) [22], together with a standardized Mandarin language assessment based on local norms [23]. Children with an IQ > 70 and a language delay of at least one year in grammar, comprehension, expression, or communication were considered eligible for DLD. Children under 6 years completed the Children Neuropsychological and Behavior Scale (CNBS) [24]; those with a nonverbal developmental quotient (DQ) > 70 and a language DQ ≤ 70, or clear language delay on the standardized Mandarin assessment, were considered for DLD. Final diagnoses were made by a senior developmental-behavioral pediatrician and a research psychologist based on developmental history, parent interview, clinical observation, and DSM-5 criteria. Typically developing (TD) children were recruited from routine health examinations. They had no parental concerns regarding speech or language development and showed age-appropriate cognitive performance (IQ or nonverbal DQ > 70) on the WISC-VI (≥6 years) or CNBS (<6 years). Informed consent was obtained from legal guardians, and the study was approved by the hospital’s Institutional Review Board.

### 2.2. Experimental Procedure

**Figure 1:**
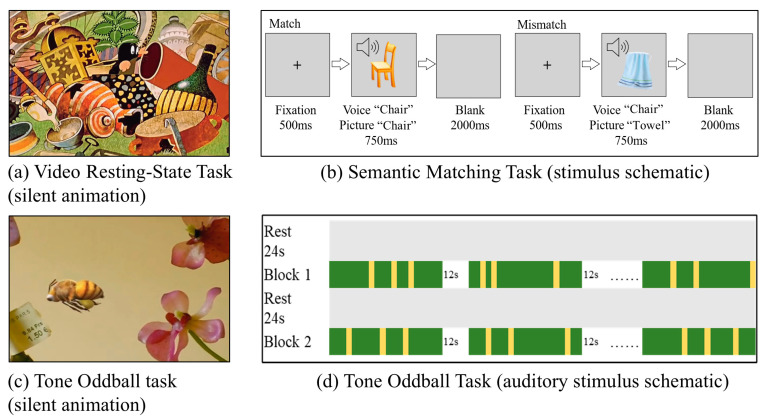
Schematic of EEG Tasks. In panel (**d**), green indicates presentation of the standard stimulus, yellow denotes the deviant stimulus, and a silent animation is displayed during the 24-s rest period and the 12-s inter-trial interval.

Upon arrival, guardians provided informed consent, and each child’s head circumference was measured to select the appropriate EGI cap. After a brief practice to ensure task comprehension, EEG recordings proceeded in a fixed sequence: (1) a 6 minutes resting-state session, (2) a 10 minutes semantic matching task (4 blocks), and (3) a 10 minutes tone oddball task. See Figure 1 for Schematic of EEG Tasks. Impedances were maintained below 50 kΩ throughout.

For the Video Resting-State Task, we used a passive viewing paradigm [25] to enhance compliance and reduce motion artifacts in young children, who are often unable to remain still during traditional eyes-open or eyes-closed resting recordings. We presented a carefully edited, emotionally neutral, silent animated video (“The Little Mole,” 6 min) to maintain natural gaze. Participants sat comfortably and watched the silent animation for the duration of the recording. This video-based paradigm is widely used in EEG studies of preschool-aged children [25] as a “rest-like” low-demand condition that improves data quality and reduces behavioral variability. For clarity, we refer to this passive viewing condition as resting state (i.e., video resting state) throughout the manuscript.

For the Semantic Matching Task, a sound–picture matching paradigm was used to assess semantic integration. The stimulus set consisted of 144 congruent pairs (e.g., “apple”–apple) and 36 incongruent pairs (e.g., “apple”–car), presented pseudo-randomly with an incongruency probability of 20%. Each trial began with a 500 ms fixation cross, followed by the simultaneous auditory and visual stimulus presentation for 750 ms, and a 2000 ms blank interval. Participants passively viewed and listened to the stimuli without providing any overt response. The 160 trials were divided into four blocks, separated by 15 s rest periods during which a silent animation was shown.

For the Tone Oddball Task, auditory discrimination and attention were measured using a syllable oddball paradigm. Two Mandarin syllables—“衣” (yī; standard) and “椅” (yǐ; deviant)—were presented at a comfortable listening level, each with a duration of approximately 1000 ms. The paradigm consisted of one initial mini block of 20 standard trials, followed by eight mixed mini blocks of 20 trials each (17 standards and 3 deviants; 15% deviant probability). Short silent-animation intervals (12 s) were inserted between mini blocks to help maintain participants’ attention. The 180 trials were divided into two blocks, separated by 24 s rest periods during which a silent animation was shown.

### 2.3. EEG Data Acquisition and Preprocessing

EEG was recorded with a 64-channel EGI HydroCel GSN system (sampling rate = 1000 Hz; online reference = Cz). Impedances were kept below 50 kΩ. Data were processed offline in MATLAB R2024b using EEGLAB v2024.2 [26] and the RELAX artifact reduction pipeline [27,28].

All preprocessing steps and artifact-rejection thresholds followed the default parameters of the RELAX automated EEG preprocessing pipeline and its developmental extension, RELAX-jr, which were specifically designed and validated for pediatric EEG recordings. Signals were high-pass filtered at 0.25 Hz, low-pass at 80 Hz, and notch-filtered at 50 Hz. The PREP pipeline identified noisy channels, and artifacts were removed using sequential Multi-channel Wiener Filtering (MWF) and wavelet-enhanced ICA (wICA). Artifact thresholds included drift (8 MADs), voltage (20 MADs; ±500 µV; 8 SDs), and muscle power slope (−0.59). Channels contaminated in >5% of data were rejected (maximum 30% of total). Removed channels were reconstructed via spherical interpolation to preserve scalp topology.

### 2.4. Data Analysis

#### 2.4.1. Resting-State Analysis

Preprocessed data were segmented into 4 s epochs and transformed using a multitaper FFT (1–40 Hz, Hanning window). Resting-state functional connectivity features were derived from debiased weighted Phase Lag Index (dwPLI) matrices computed across all electrode pairs. For each participant, dwPLI values were averaged within predefined canonical frequency bands: delta (1–3 Hz), theta (4–7 Hz), alpha (7–10 Hz), beta (11–20 Hz), and gamma (21–40 Hz). These band-specific connectivity matrices were used as input for group-level statistical comparisons.

Microstate analysis was conducted using MICROSTATELAB v2.1 [29]. Global Field Power (GFP) peaks were clustered into seven microstate classes following [14]. Microstate features were quantified by extracting a comprehensive set of temporal parameters for each microstate class. These included mean duration, temporal coverage (percentage of total recording time), occurrence rate (number of occurrences per second), transition probabilities between all pairs of microstates, and the standard deviation of GFP at time points assigned to each microstate. Together, these parameters were used to characterize the stability and switching dynamics of large-scale neural states and compared between groups via Mann–Whitney U tests.

#### 2.4.2. Semantic Matching Task Analysis

Preprocessed data were epoched (−200 to 1000 ms), baseline-corrected, and artifact-rejected (>100 µV) using RELAX pipeline. ERP features were quantified as mean amplitudes within predefined time windows of interest at selected midline electrodes (Fz, FCz, Cz, CPz, and Pz). Early perceptual processing was examined in the 100–300 ms window, while later semantic-related activity was quantified within the extended N400 time window (500–700 ms), based on prior developmental ERP literature. Group comparisons were conducted using non-parametric cluster-based permutation tests (FieldTrip [30]), with clusters defined across spatially neighboring electrodes. This approach controls the family-wise error rate across electrodes and time points, providing correction for multiple comparisons.

#### 2.4.3. Tone Oddball Task Analysis

Time–frequency features were quantified by averaging baseline-corrected power values within predefined frequency bands and post-stimulus time windows. Epochs (−1000 to 1500 ms) were analyzed using Morlet wavelets (3–40 Hz, 1 Hz steps, width = 7). Power was baseline-corrected (−0.8 to −0.2 s) and converted to dB. Analyses focused on theta (4–7 Hz), alpha (7–10 Hz), beta (11–20 Hz), and gamma (21–40 Hz) bands, commonly linked to attention, inhibitory control, and large-scale neural synchronization. The extracted features for each participant included the mean baseline-corrected power in each frequency band and post-stimulus time window at each electrode. Group comparisons used non-parametric cluster-based permutation tests.

#### 2.4.4. Machine Learning Classification

To examine the discriminative power of electrophysiological features, only features that showed significant group differences were selected for classification. Specifically, resting-state microstate metrics included transition probability and GFP standard deviation measures that differed between groups. For the semantic matching task, P1 and N2 peak amplitudes and latencies were extracted at electrodes O1, O2, and Oz. For the tone oddball task, time–frequency features consisted of the mean power in the 7–10 Hz (alpha) band within the 300–500 ms window at electrodes AF3, F3, F5, and FC5. All selected features were standardized (z-score normalization) and submitted to PCA before entering the machine learning classifiers.

Dimensionality reduction was performed via PCA (95% variance retained), followed by a 70/30 train–test split. Model training and optimization will be performed on the training set using a 5-fold stratified cross-validation procedure, with final model performance subsequently evaluated on the held-out test set. The classifiers to be evaluated include Linear Discriminant Analysis (LDA), Support Vector Machine (SVM), and K-Nearest Neighbors (KNN). For SVM, the BoxConstraint was set to 1, and KernelScale was automatically determined; KNN was configured with 10 neighbors, cosine distance, and equal distance weighting; LDA employed linear discriminant analysis. All classifiers included standardization of predictors. Classification performance was assessed using several standard metrics: Accuracy, reflecting the proportion of all correct classifications; Precision, the proportion of predicted positive cases that are actual positives; Recall, the proportion of actual positive cases correctly identified; and the F1-Score, the harmonic mean of Precision and Recall, which provides a comprehensive measure of model robustness. Model performance was evaluated using 5-fold cross-validation, where 4/5 of the data were used for training and 1/5 for validation in each fold. Predictions across folds were combined to calculate overall validation accuracy, ensuring robust estimation of classifier performance while mitigating overfitting.

## 3. Results

### 3.1. Resting-State Neurodynamic Reveal Atypical Brain Network Organization in DLD

#### 3.1.1. Frequency-Specific Functional Connectivity Alterations

**Figure 2:**
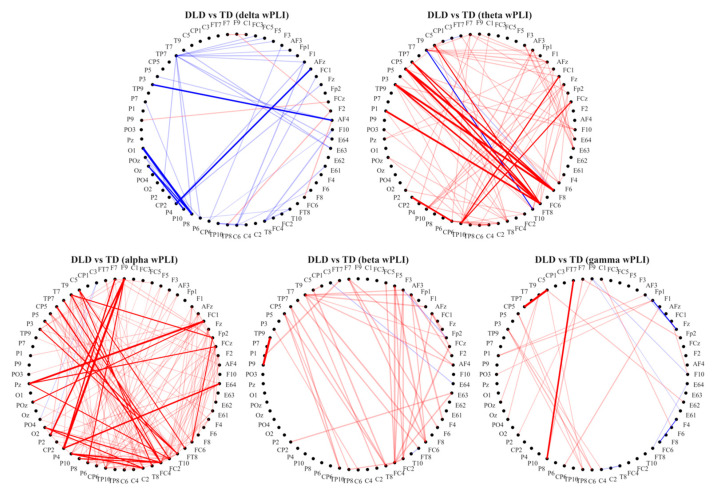
Between-group differences in resting-state functional connectivity across different frequency bands. Note. Red lines indicate stronger phase synchrony in the DLD group compared to the TD group (DLD > TD), while blue lines indicate weaker synchrony (DLD < TD). Darker lines represent connections with uncorrected *p* < 0.001, and lighter lines represent uncorrected *p* < 0.01. The thickness of the line corresponds to the magnitude of the T-value.

Resting-state functional connectivity, estimated via dwPLI, revealed marked frequency-specific differences between groups (Figure 2, Table 1). Compared with TD children, the DLD group exhibited reduced connectivity (hypoconnectivity) in both the delta (1–3 Hz) and gamma (21–40 Hz) bands. Delta-band hypoconnectivity was mainly localized to posterior occipito-parietal regions, whereas gamma-band deficits involved short-range frontal connections. In contrast, hyperconnectivity was observed across mid-frequency bands (theta, alpha, beta), forming extensive long-range connections from right temporo-parietal and frontal regions to distributed cortical areas, as well as enhanced midline synchronization. This pattern suggests a frequency-dependent reorganization of large-scale brain networks in DLD, characterized by weakened low- and high-frequency coupling but excessive synchronization at intermediate frequencies.

#### 3.1.2. Altered Microstate Dynamics

**Figure 3:**
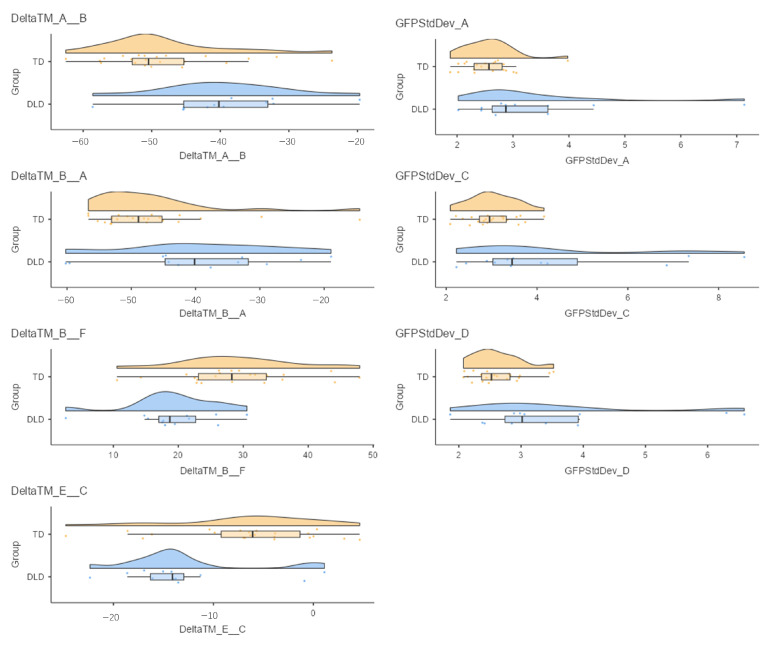
Between-group differences in microstate parameters in children. Note. DeltaTM refers to the difference between the observed transition probability and the expected transition probability, where a smaller value indicates a lower transition probability. GFPStdDev refers to the standard deviation of the Global Field Power (GFP) at the time points assigned to each microstate template; a smaller value indicates less fluctuation in the activity strength of that state. Yellow dots indicate the values of the metric for the TD group, whereas blue dots indicate the values for the TD group.

Non-parametric group comparisons (Mann–Whitney U tests) revealed significant between-group differences in microstate transition probabilities and network stability (Global Field Power standard deviation, *p* < 0.05, uncorrected, Figure 3), while mean duration, coverage, and occurrence rates did not differ significantly.

Children with DLD showed increased bidirectional transitions between Microstate A (auditory network) and Microstate B (primary visual network) (ΔTM_A → B: −39.4 vs. −48.1; ΔTM_B → A: −39.3 vs. −47.1), suggesting atypical cross-modal switching. Conversely, transitions from Microstate B to F (fronto-parietal control network; 19.2 vs. 29.1) and from Microstate E (higher-order visual) to C (default mode; −13 vs. −6.66) were significantly reduced, indicating reduced integration between perceptual and control networks.

Additionally, DLD children exhibited higher GFP variability, reflecting increased temporal instability of global neural states. Elevated GFP standard deviations were observed for Microstate A (3.33 vs. 2.57), Microstate C (4.33 vs. 3.03), and Microstate D (3.55 vs. 2.61). Together, these findings indicate less stable and less efficient large-scale neural dynamics in DLD.

### 3.2. Task-Evoked Neural Responses Reveal Deficits in Language and Auditory Processing

#### 3.2.1. Semantic Matching Task: Attenuated ERP Components

**Figure 4:**
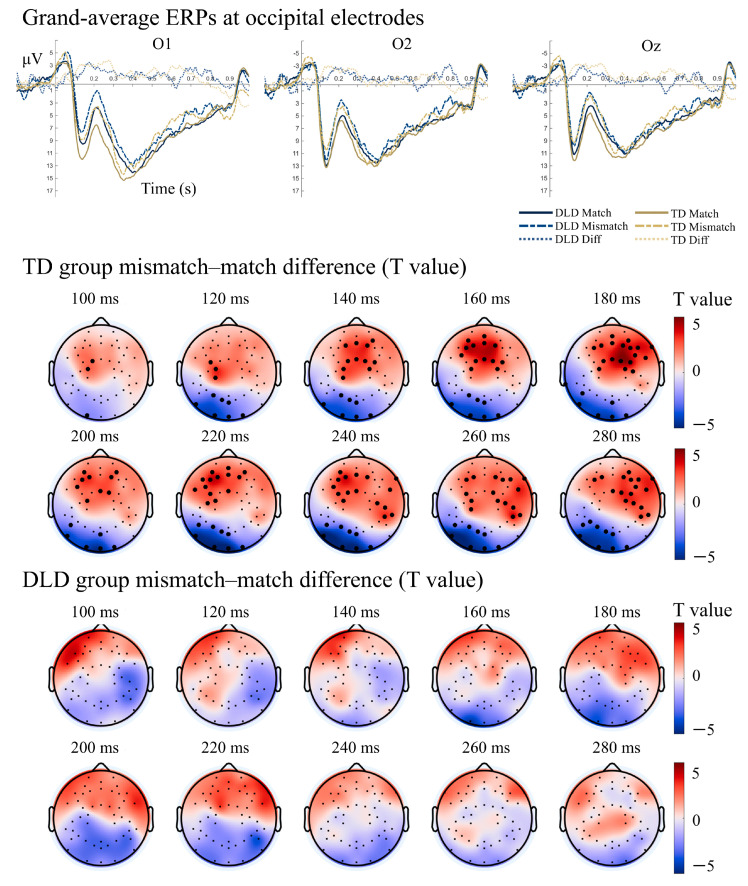
Grand-average ERPs at occipital electrodes and the t-value topography of the group difference wave (100–300 ms) during the semantic matching task for TD and DLD children. Note. The yellow solid line represents the grand-average ERP for the match condition in the TD group; the yellow dashed line represents the grand-average ERP for the mismatch condition in the TD group; the light yellow dashed line represents the grand-average difference wave for the TD group. The blue solid line represents the grand-average ERP for the match condition in the DLD group; the blue dashed line represents the grand-average ERP for the mismatch condition in the DLD group; the light blue dashed line represents the grand-average difference wave for the DLD group. Bolded dots indicate electrodes where the difference wave (mismatch—match) showed a significant difference after correction (*p_FWE_* < 0.05).

During the early processing stage (100–300 ms) of the semantic matching task, differences between match and mismatch conditions emerged at occipital electrodes in both the TD and DLD groups (P1 match amplitude > P1 mismatch amplitude; N2 mismatch amplitude > N2 match amplitude). However, these differences were significant, more widespread, and sustained in the TD group (Figure 4). Conversely, although the DLD group exhibited a similar trend, this effect was not significant after correction (Figure 4).

**Figure 5:**
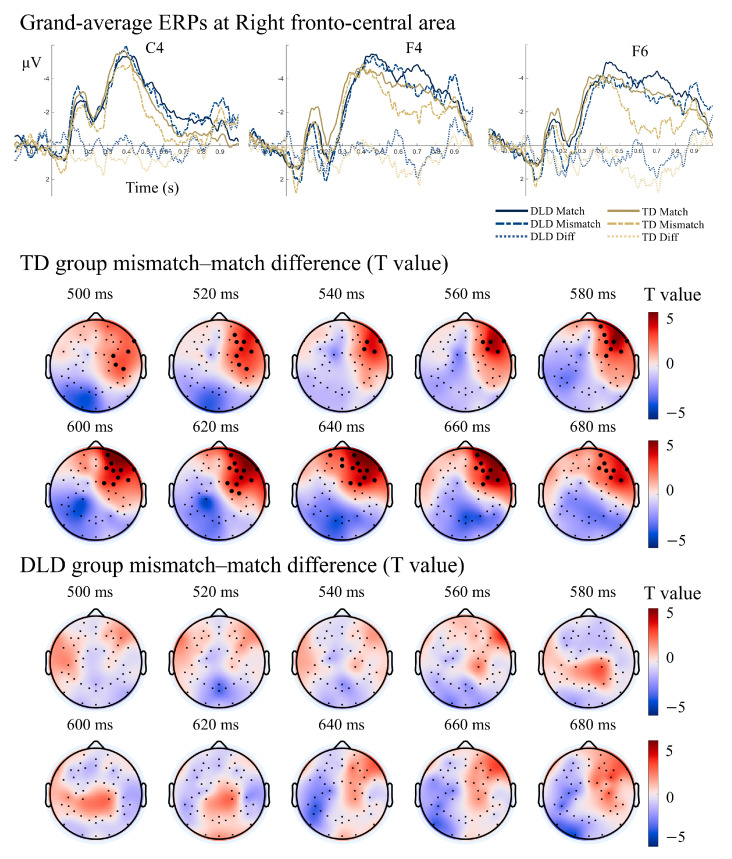
Grand-average ERPs at bilateral anterior electrodes and the t-value topography of the group difference wave (500–700 ms) during the semantic matching task for TD and DLD children. Note. The yellow solid line represents the grand-average ERP for the match condition in the TD group; the yellow dashed line represents the grand-average ERP for the mismatch condition in the TD group; the light yellow dashed line represents the grand-average difference wave for the TD group. The blue solid line represents the grand-average ERP for the match condition in the DLD group; the blue dashed line represents the grand-average ERP for the mismatch condition in the DLD group; the light blue dashed line represents the grand-average difference wave for the DLD group. Bolded dots indicate electrodes where the difference wave (mismatch—match) showed a significant difference after correction (*p_FWE_* < 0.05).

Within the N400 window (300–600 ms), neither group showed a significant mismatch–match difference. However, during the extended N400 period (500–700 ms), TD children demonstrated a significant right fronto-central difference wave, absent in the DLD group (Figure 5). These results suggest delayed and attenuated semantic integration processes in DLD, particularly in later stages of meaning retrieval.

#### 3.2.2. Tone Oddball Task: Impaired Oscillatory Power Modulation

No clear mismatch negativity (MMN) was detected in either group between 100 and 200 ms. No significant group differences were detected in the time–frequency analysis after correction for multiple comparisons (*p_FWE_* < 0.05). Given the exploratory nature of this study, we also report the results at an uncorrected threshold of *p* < 0.001. At this threshold, time–frequency analysis revealed that TD children exhibited stronger deviant–standard power differences, forming a spatio–temporal–spectral cluster. This effect was primarily localized to the 7 Hz band at channels AF3 and F3 (300–400 ms), the 8 Hz band at channels AF3 and F5 (400–500 ms), and the 9 Hz band at channel AF3 (400–500 ms).

**Figure 6:**
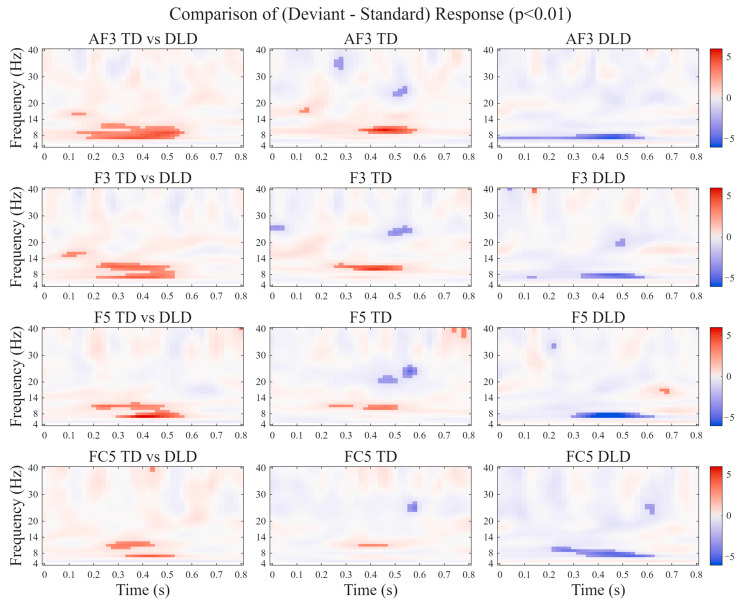
T-value topography maps of the deviant–standard time-frequency difference at left fronto-central sites during the tone oddball task. Note: The first column displays the T-value topography map of the between-group comparison (TD vs. DLD) of the deviant–standard time-frequency difference. The second column displays the T-value map for the deviant–standard time-frequency difference within the TD group. The third column displays the T-value map for the deviant–standard time-frequency difference within the DLD group. Each row represents the results at channels AF3, F3, F5, and FC5, respectively.

Within-group comparisons showed opposite oscillatory patterns. TD children displayed event-related synchronization (ERS) in the 9–11 Hz (alpha) band, indicating enhanced neural engagement to deviant stimuli. In contrast, DLD children showed event-related desynchronization (ERD) in the 7–8 Hz (high-theta/low-alpha) range, reflecting reduced attentional resource allocation and weaker top-down modulation (Figure 6). To more clearly illustrate these trends, Figure 6 presents the time–frequency results at an uncorrected threshold of *p* < 0.01.

### 3.3. Classification Accuracy Using a Machine Learning Model

**Figure 7:**
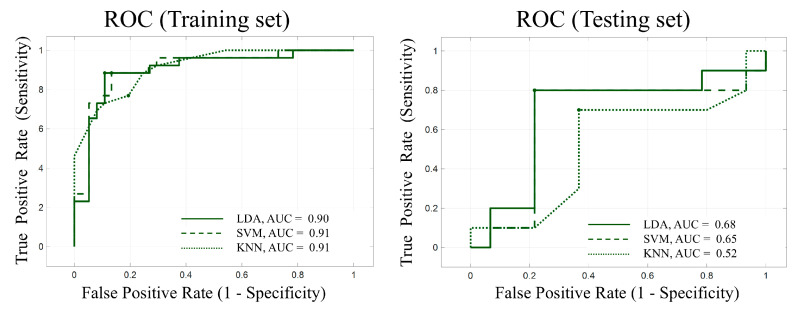
ROC Curves for the Classification Models Based on EEG features from Three Tasks.

To evaluate the discriminative potential of electrophysiological features, three classifiers—Linear Discriminant Analysis (LDA), Support Vector Machine (SVM), and K-Nearest Neighbors (KNN)—were trained (Figure 7, Table 2).

Among them, LDA achieved the highest training F1-score (88.51%) and AUC (0.90) but declined on the test set (F1 = 80.0%, AUC = 0.68), indicating mild overfitting. Similarly, linear SVM and cosine KNN achieved training F1-scores of 88.37% and 76.92%, respectively, but demonstrated reduced test performance. Across models, results consistently suggest moderate classification accuracy with limited generalizability, underscoring the need for larger samples and model regularization. Given the small sample size and limited test-set size, the observed test performance should not be interpreted as reliable evidence of discriminative power, but rather as an unstable estimate reflecting exploratory model behavior.

## 4. Discussion

The present study was designed as an exploratory investigation to evaluate the feasibility of integrating multi-level electrophysiological features—spanning resting-state and task-evoked neural dynamics—to differentiate children with developmental language disorder (DLD) from their typically developing (TD) peers. Rather than establishing a diagnostic classifier, our primary objective was to examine whether resting-state neural architecture and task-specific computations jointly contain discriminative information, and to identify the analytical challenges inherent in such a high-dimensional framework. The findings offer convergent support for this premise: DLD is associated with an atypical foundational neural state that cascades into altered task-related computations. However, while these multimodal signatures show promise for group-level differentiation, their limited generalizability underscores the challenge of achieving clinically robust prediction.

### 4.1. Atypical Intrinsic Network Architecture in DLD

Our first hypothesis—predicting an atypical intrinsic neural organization in DLD—was supported by both functional connectivity and microstate analyses. The connectivity results revealed not a uniform deficit but a complex pattern of spectral imbalance. Specifically, DLD children exhibited reduced connectivity in the slow-wave delta and fast-wave gamma bands, alongside widespread hyper-connectivity across the mid-frequency theta, alpha, and beta ranges.

This pattern of frequency-specific imbalance aligns with prior neurophysiological evidence of disrupted functional coupling in neurodevelopmental disorders. Mid-band hyper-connectivity—particularly involving right-hemisphere and midline structures—resembles previous findings in ADHD and ASD [31,32] and may reflect an inefficient, or “noisy,” neural architecture, possibly resulting from impaired synaptic pruning or excessive compensatory engagement. In contrast, posterior delta hypo-connectivity and frontal gamma reduction may, respectively, index weakened large-scale integrative communication and compromised local cortical computations, both of which are critical for language and executive functioning [33].

From a mechanistic perspective, such spectral imbalance may reflect multiple, non-mutually exclusive neurobiological processes, including altered excitation–inhibition balance, atypical maturation or myelination of long-range pathways, and disrupted thalamocortical coordination. Each of these mechanisms has been shown to differentially affect oscillatory activity across frequency bands and to constrain efficient communication across temporal and spatial scales, providing a biologically plausible framework for interpreting the observed connectivity profile in DLD.

The microstate findings indicate that children with DLD exhibit atypical modulation of neural activity during rest. Although static temporal parameters (mean duration, coverage) were comparable between groups, the DLD group exhibited significantly greater instability—indexed by increased GFP standard deviation—in microstates A, C, and D, which have been putatively associated with auditory, default mode, and dorsal attention networks in prior studies. This pattern may suggest that the core functional systems supporting auditory perception, self-referential cognition, and goal-directed attention are less stable or more variable in DLD. Similar microstate instability has been observed in related developmental disorders such as dyslexia [34] and ASD [35], implicating a broader phenomenon of impaired neural self-organization. Furthermore, the altered transition probabilities—particularly excessive oscillation between auditory and visual states (A ↔ B) and reduced switching toward higher-order control states (B → F, E → C)—indicate diminished network flexibility or a “sticky” functional architecture [36]. Such rigidity may mechanistically underlie the attentional and cognitive control difficulties often reported in DLD. These functional interpretations are necessarily grounded in prior microstate literature linking canonical microstate classes to large-scale networks, as source localization was not performed in the present study. Accordingly, network-level labels should be interpreted as putative rather than definitive.

Finally, it is important to clarify the nature of the resting-state recordings used in this study. EEG data were acquired during passive viewing of a silent animated video rather than during canonical eyes-open or eyes-closed rest. Although sustained visual input may engage visual networks to some extent, this video-based paradigm is widely used in preschool-aged children as a low-demand, compliance-optimized condition that minimizes motion artifacts and behavioral variability. As such, the present results are best interpreted as reflecting intrinsic network organization during a rest-like passive viewing state, rather than pure resting-state activity.

### 4.2. Propagation to Task-Based Processing Deficits

Contrary to our initial hypothesis, we did not observe significant reductions in P300 or MMN amplitudes in children with DLD during task-based paradigms. However, children with DLD exhibited other task-related abnormalities.

In the semantic matching task, both groups exhibited early visual ERP differences (P1/N2) between match and mismatch conditions. In DLD, this effect was weaker and more spatially limited, suggesting less reliable early perceptual processing. Neither group showed a canonical N400 mismatch effect (300–600 ms), possibly due to task-specific factors. During our child-friendly paradigm, the task was passive, mismatch trials were infrequent (20%), and no explicit behavioral decision was required. Under low attentional and low expectancy-violation demands, classic N400 effects can be attenuated because semantic prediction errors are smaller and participants may not consistently allocate resources to evaluate incongruency on each trial. However, TD children showed a late positive component (500–700 ms) over right fronto-central electrodes, which was absent in DLD. In TD, sustained late activity likely reflects ongoing semantic reanalysis or integration closure [37]. The lack of this response in DLD may indicate difficulty maintaining or updating semantic representations, consistent with previous reports of reduced or delayed N400 amplitudes [38,39]. Future work should increase expectancy-violation strength (e.g., higher mismatch probability), incorporate an explicit congruency decision and/or child-appropriate attention checks, and systematically vary task demands to more reliably elicit canonical N400 responses and disentangle N400-linked semantic prediction/integration from later evaluative–reanalysis processes.

In the tone oddball task, neither group exhibited a classical MMN in the ERP domain, consistent with mixed findings in the DLD literature [19,40]. Time–frequency analyses, however, revealed a clear group difference. TD children showed significant alpha-band event-related synchronization (ERS) at left frontal electrodes (400–500 ms), reflecting efficient top-down inhibitory control [41]. In contrast, the DLD group exhibited high-theta/low-alpha event-related desynchronization (ERD) over the same regions and time window, reflecting reduced inhibition and less efficient attentional modulation [42]. This ERS–ERD polarity reversal provides a compelling neural marker of inefficient resource allocation in DLD, consistent with a “noisy” and unstable baseline state that fails to support optimal task engagement.

### 4.3. Feasibility and Challenges of a Multi-Level Classifier

Our third hypothesis concerned the feasibility of integrating multi-level neural features within a machine learning framework. Accordingly, the classification analyses were intended as a proof-of-concept rather than a test of robust discriminative performance. The classification analyses provided two exploratory observations. First, the training set performance (e.g., LDA F1 = 88.51%, AUC = 0.90) suggests that combining resting-state and task-based features is potentially informative, although these results should be interpreted with caution. Similar approaches in EEG- and fNIRS-based studies of neurodevelopmental disorders have reported comparable accuracies [43,44]. As anticipated, performance did not generalize well to an independent test set (F1 = 80.00%, AUC = 0.68). This overfitting highlights a common limitation of small-sample, high-dimensional neuroimaging studies. Rather than indicating failure, it underscores a methodological consideration: models trained on limited or noisy data may capture sample-specific variance rather than generalizable neurobiological signatures [45].

Taken together, the machine learning analyses do not demonstrate robust or generalizable classification of DLD at this stage. Instead, they illustrate the potential informativeness of combining resting-state and task-based neural features, while highlighting the substantial methodological challenges posed by small-sample, high-dimensional neurodevelopmental data. Larger, independent datasets and more rigorous validation frameworks will be required to determine whether such approaches can yield clinically meaningful biomarkers.

### 4.4. Implications for Neural Models of Language

Our multi-level EEG findings can be interpreted within contemporary neurobiological frameworks of language, while remaining cautious about the inferential limits of scalp-level measures. Cortical organization accounts proposed that successful language comprehension depends on coordinated interactions across distributed fronto-temporal systems, with partially dissociable contributions of inferior frontal and posterior temporal regions to syntactic/structural processing and its interface with memory and control demands [8]. A recent review argued that a “core” left-lateralized language network constitutes a functionally distinct system embedded within a broader landscape of perceptual–motor and domain-general networks that interact with it during comprehension and production [7]. Within this perspective, the group differences between DLD and TD we observed in baseline (rest-like passive viewing) network organization and in task-evoked responses are compatible with altered efficiency in the coordination between language-relevant computations and broader control/attentional architecture, rather than pointing to a single isolated deficit within a putatively language-specific module.

Oscillation-based proposals provide an additional computational lens. Models such as ROSE emphasize that linguistic representations and operations can be framed in terms of rhythmic coordination across neural ensembles and hierarchical timing mechanisms [6]. From this viewpoint, the frequency-specific imbalance we observed in baseline functional organization (together with atypical task-related dynamics) is broadly compatible with disrupted temporal coordination that may reduce the stability or efficiency of higher-order updates. However, our present results are not specific enough to adjudicate among competing accounts, underscoring the importance of task designs that systematically manipulate expectancy violation and attention.

### 4.5. Limitations and Strengths

The primary limitation of this study lies in its modest sample size, particularly for the DLD group—a common challenge in pediatric clinical neuroimaging due to stringent diagnostic criteria and compliance constraints. In addition, the cross-sectional nature of the study limits inferences about developmental trajectories or causal mechanisms. Nevertheless, the sample size is comparable to or exceeds many prior EEG and ERP studies on developmental language disorders. To mitigate potential bias, we employed rigorous non-parametric, cluster-based permutation tests that effectively control false positives in high-dimensional data. Moreover, the within-subject contrasts in task paradigms enhanced statistical power by reducing inter-individual variability. Importantly, the converging evidence across three independent analytical levels—resting-state connectivity, microstate dynamics, and task-evoked responses—supports the reliability of the observed group differences. Still, the machine learning findings should be regarded as preliminary, emphasizing the need for larger, longitudinal cohorts to validate classification performance and explore heterogeneity within DLD.

## 5. Conclusions

This study identifies a multi-level pattern of neural atypicalities in children with DLD, spanning resting-state network organization and task-evoked processing. DLD was associated with atypical intrinsic connectivity patterns and reduced stability of core functional networks, alongside weaker semantic processing and inefficient oscillatory dynamics during auditory attention. These multi-level features show clear group distinctions, suggesting potential utility for future biomarker development. However, their limited generalizability highlights the need for larger samples and cross-site validation before clinical implementation. Overall, our findings provide a neurophysiological framework that may support earlier detection and more targeted intervention strategies for developmental language disorder.

## Figures and Tables

**Table 1:** Between-group differences in functional connectivity during the resting-state EEG task.

Band	Channel A	Channel B	T	*p*
delta	O1	P8	−3.92	0.0001
(1–3 Hz)	AFz	P4	−3.28	0.0008
	AF4	P3	−3.22	0.0010
	Oz	P10	−3.07	0.0007
	Oz	P8	−2.63	0.0003
theta	CP5	F8	4.57	0.0002
(4–7 Hz)	P3	F8	4.33	0.0003
	CP5	FT8	4.27	0.0001
	P1	FT8	4.15	0.0002
	CP2	CP6	4.02	0.0003
	P3	FT8	3.85	0.0001
	TP7	F8	3.49	0.0007
	TP7	FT8	3.48	0.0003
	FC1	TP10	3.30	0.0008
	FCz	TP10	3.15	0.0009
	C5	FT8	3.10	0.0007
	T9	T10	−2.97	0.0004
alpha	F9	P4	5.48	0.0001
(7–10 Hz)	FC1	Pz	5.02	0.0001
	P4	E64	4.48	0.0003
	P10	FC4	4.46	0.0002
	F9	P2	4.45	0.0002
	TP7	FC4	4.27	0.0003
	P10	C2	4.16	0.0002
	F7	P4	4.16	0.0001
	P5	T10	3.91	0.0002
	Fp2	T9	3.75	0.0002
	O2	FC4	3.75	0.0003
	O2	C2	3.72	0.0009
	P8	FC4	3.64	0.0004
	F9	P6	3.60	0.0007
	FCz	Pz	3.60	0.0010
	T7	FC2	3.58	0.0010
	P3	T10	3.58	0.0010
	FC1	POz	3.54	0.0010
	T9	FC4	3.48	0.0009
	Pz	FC2	3.47	0.0008
	C5	T8	3.46	0.0010
	C5	FT8	3.44	0.0009
	Fp2	FC6	3.35	0.0010
beta	TP9	P9	3.58	0.0002
(11–20 Hz)				
gamma	C5	TP7	4.66	0.0001
(21–40 Hz)	FT7	P8	4.01	0.0004
	FC6	F4	−2.45	0.0007
	Fz	Fp1	−2.99	0.0003

Note. Only results with uncorrected *p* < 0.001 are shown.

**Table 2:** Classification Model Performance Based on EEG Metrics from three EEG Tasks.

Classifier	Training Set				Test Set			
	Accuracy	Weighted Precision	Weighted Recall	Weighted Recall	Accuracy	Weighted Precision	Weighted Recall	Weighted Recall
LDA	88.46%	88.83%	88.46%	88.51%	80.00%	80.00%	80.00%	80.00%
SVM	88.46%	88.56%	88.46%	88.37%	80.00%	80.00%	80.00%	80.00%
KNN	76.92%	80.65%	76.92%	76.92%	70.00%	69.52%	70.00%	69.01%

## Data Availability

Due to privacy and ethical restrictions, the raw data of this study are not publicly available.
